# AlgiMatrix™ Based 3D Cell Culture System as an In-Vitro Tumor Model for Anticancer Studies

**DOI:** 10.1371/journal.pone.0053708

**Published:** 2013-01-18

**Authors:** Chandraiah Godugu, Apurva R. Patel, Utkarsh Desai, Terrick Andey, Alexandria Sams, Mandip Singh

**Affiliations:** 1 College of Pharmacy and Pharmaceutical Sciences, Florida A&M University, Tallahassee, Florida, United States of America; 2 Primary and Stem Cell Systems, Cell Systems Division, Life Technologies Corporation, Frederick, Massachusetts, United States of America; Wayne State University, United States of America

## Abstract

**Background:**

Three-dimensional (3D) in-vitro cultures are recognized for recapitulating the physiological microenvironment and exhibiting high concordance with in-vivo conditions. Taking the advantages of 3D culture, we have developed the in-vitro tumor model for anticancer drug screening.

**Methods:**

Cancer cells grown in 6 and 96 well AlgiMatrix™ scaffolds resulted in the formation of multicellular spheroids in the size range of 100–300 µm. Spheroids were grown in two weeks in cultures without compromising the growth characteristics. Different marketed anticancer drugs were screened by incubating them for 24 h at 7, 9 and 11 days in 3D cultures and cytotoxicity was measured by AlamarBlue® assay. Effectiveness of anticancer drug treatments were measured based on spheroid number and size distribution. Evaluation of apoptotic and anti-apoptotic markers was done by immunohistochemistry and RT-PCR. The 3D results were compared with the conventional 2D monolayer cultures. Cellular uptake studies for drug (Doxorubicin) and nanoparticle (NLC) were done using spheroids.

**Results:**

IC_50_ values for anticancer drugs were significantly higher in AlgiMatrix™ systems compared to 2D culture models. The cleaved caspase-3 expression was significantly decreased (2.09 and 2.47 folds respectively for 5-Fluorouracil and Camptothecin) in H460 spheroid cultures compared to 2D culture system. The cytotoxicity, spheroid size distribution, immunohistochemistry, RT-PCR and nanoparticle penetration data suggested that in vitro tumor models show higher resistance to anticancer drugs and supporting the fact that 3D culture is a better model for the cytotoxic evaluation of anticancer drugs in vitro.

**Conclusion:**

The results from our studies are useful to develop a high throughput in vitro tumor model to study the effect of various anticancer agents and various molecular pathways affected by the anticancer drugs and formulations.

## Introduction

Traditionally, most in-vitro cell cultures are grown in two dimensional (2D) environments. In mammalian tissues and cells connect not only to each other, but also to support structures called extracellular matrix (ECM). The cells grow within an organized three dimensional (3D) matrix and their behavior is dependent upon interactions with immediate neighbors and the ECM [1], [2], [3]. Integrin surface receptors anchor their bearers to the ECM, and mediate biochemical signal interpretation that leads cells to undergo differentiation, apoptosis, proliferation, or invasion [4]. Though, most cell cultures are grown in 2D environments, they do not accurately recapitulate the structure, function, or physiology of living tissues [1], [5].

Cancer researchers typically rely on 2D in-vitro studies and small animal models to study the complex mechanisms of tumor angiogenesis, invasion, and metastasis [6]. The cell-cell and cell-matrix interactions observed during in vivo tumor progression cannot be studied in 2D models while, 3D models are capable of mimicking these conditions [7]. The 3D cultures may play a potential role in cancer drug discovery due to the lack of relevant preclinical models and advantages over 2D cultures [8]. Although, animal models are accurate representative of tumor environment, they are considerably less amenable to large-scale screening.

Matrix-based 3D in-vitro culture models are increasingly becoming essential tools in cancer research as they allow cell responses that more closely mimic events occurring in-vivo during cancer formation and progression [7], [9]. Novel strategies are being applied for creating better in-vitro models that simulate in-vivo conditions for testing the efficacy of anticancer drugs [10]. They provide a pathophysiological context that more accurately replicates the solid cancer microenvironment compared to monolayer cultures in 2D system [7]. The pre-vascularized initial stages of solid tumor growth can be characterized by identifiable criteria within the tumor microenvironment, including an uninhibited 3D proliferative capacity, regions of hypoxia surrounding a necrotic core and activation of genetic factors that lead to the recruitment of local endothelial cells for self-sustaining angiogenesis [6].

3D cell culture models create a pragmatic microenvironment and mimic an in vivo system, which helps to understand cell-cell interactions [11]. Cells cultured in a 3D in vitro environment have the ability to acquire phenotypes and respond to stimuli analogous to in vivo biological systems. This approach can be applied to the development of a physiologically relevant in vitro tumor model. The universal acceptance of the 3D paradigm is currently hindered by the lack of a suitable biocompatible material that offers ease of use, experimental flexibility, and a seamless transition from in-vitro to in-vivo applications [12]. Among 3D cultures, 3D alginate scaffold has advantages being an animal-free product with significant stability at room temperature [13]. 3D alginate scaffold is a non-toxic and biodegradable ready-to-use sponge made from lyophilized alginate gel which supports a cell culture model resembling normal cell characteristics and morphology. Translucent multicellular spheroids can be easily visualized under the microscope to monitor the growth characteristics. AlgiMatrix™ is a chemically defined, highly porous (>90%) 3D scaffold and recovery from 3D alginate scaffold is achieved with the use of dissolving buffer, a non-enzymatic solution which dissolves the scaffold within a few minutes but leaves the cellular aggregates intact for further processing and/or analysis. Spheroid formation is restricted by the fixed pore sizes (50–150 µm) within the sponge thereby preventing the development of hypoxic cores. Long-term viability, cellular toxicity measurements, & ultrastructural analysis demonstrate that the 3D alginate scaffold environment promotes a healthier phenotype than the conventional sandwich culture. It is expected that the development of 3D tumor models will reduce animal testing, yield more predictive data, improve cell culture efficiency, reduce cost and time to identify new drug candidates and reduce development time to market [5], [14], [15]. It is our hypothesis that the use of an alginate based 3D scaffold lung tumor model will simulate in vivo conditions to screen the efficacy of drug candidates and will be more effective than the use of traditional monolayer 2D cultures. The pre-vascularized initial stages of solid tumor growth can be generated in the 3D in vitro tumor models. Further, the hypoxia regions surrounding necrotic cores can also be simulated in 3D models by growing spheroids to above 200 µm sizes.

In the present study we have optimized and developed the in vitro alginate scaffold 3D tumor model using H460, A549, and H1650 non small cell lung cancer (NSCLC) cells. Anticancer effects of various chemotherapeutic agents were studied and IC_50_ values were compared with the conventional 2D cell culture models. H1650 NSCLC stem cells were also developed as 3D in -vitro tumor models and the effect of various anticancer drugs were studied and compared with 3D H1650 parental cells and stem cell monolayer cultures. Further, we also studied the uptake pathways for free drug and nanoparticles containing their payloads into the spheroids. Finally, the effect of anticancer drugs on apoptosis induction and other antiapoptotic markers were studied and compared with the 2D cell culture systems.

## Materials and Methods

### Materials

AlgiMatrix™ 3D culture supplies and alamarBlue® dye were obtained from Life Technologies Corporation, Carlsbad (Carlsbad, California 92008). The human Non Small Cell Lung Cancer (NSCLC) cell lines H460, A549, H1650, and H1650 stem cells were obtained from American Type Culture Collection (Manassas, VA 20110). DIO oil was purchased from Molecular Probes; Docetaxel, Doxorubicin, Cisplatin, Gemcitabine, 5-Fluorouracil, and Camptothecin were obtained from Sigma Chemicals. St. Louis, MO. All other chemicals were either reagent or tissue culture grade.

### Incorporation of cancer cells into 3D matrix

In 96 or 6 well 3D alginate scaffold plates, cells with density range of 1–35×10^3^ or 0.05–6×10^6^ were incorporated into 3D alginate scaffold in 100 or 2000 µL of media, respectively. After 20 min, another 200 or 3000 µL of media was added and cells were grown in incubator at 37°C with 5% CO_2._ Media was changed every 48 h. Drug treatment was started at 7, 9 and 11 days post cell seeding. At each treatment time point, drug was exposed for 24 h, followed by a 24 h wash period. At the end of the treatment, cell viability was estimated by alamarBlue® assay in the intact matrix.

### Development and Optimization of In-Vitro 3D Lung Tumor Model

#### A) 3D alginate scaffold in 6 well plates

NSCLC cell lines such as H460, A549 and H1650 were incorporated in each well of the 6 well plates at different cell concentrations (0.05, 0.10, 0.15, 0.25, 0.50, 0.75, 1.0, 1.25, 1.50, 3 & 6 million cells/well) to determine the optimal seeding density and time for the formation of spheroids. Then 2 ml of cell suspension containing firming buffer was added to the 3D alginate scaffold cultures system. The growth of tumor spheroids was assessed by observing formation of the spheroids in 3D alginate scaffold well. At 4 d, 9 d and 13 d post cell seeding, images were taken using an inverted microscope (Motic AE 31, BC, Canada), the size and number of spheroids were determined. Based on results, cell concentrations of 0.15 & 0.25 million cells per well of 6-well plate were used to establish alginate scaffold 3D tumor model.

#### B) 3D alginate scaffold in 96 well plates

H460, A549 and H1650 cells at the cell density range of 1–35×10^3^ cells per well were introduced into the 3D alginate scaffold in 100 µL volume. After 20 minutes, another 200 µL of media was added. Based on the initial screening on spheroid number and size distribution, 15,000 cells per well were selected for further cytotoxicity evaluations. After establishing the 96 well format 3D culture systems, anticancer effect of Docetaxel was studied with all 4 types of lung cancer cells. On 4th, 9th and 13th day post cell seeding, images were taken using an inverted microscope.

### Spheroid size and number evaluation

It is possible to observe the spheroids growing in the 3D alginate scaffold without dissolving the matrix or removing media. During 13 days of spheroid growth, the size of spheroid and number of spheroids in each well were measured or counted on an inverted microscope. From each well an average of 8–10 fields were used for these measurements. The effect of anticancer drugs on spheroids number and size distribution was studied during the anticancer evaluations.

### Efficacy of Docetaxel in alginate scaffold 3D Lung Tumor Model

For initial experiments, all the lung cancer cell types were studied with Docetaxel. Docetaxel (25–400 µM) was used to treat 3D alginate scaffolds seeded with 0.15 million and 0.25 million cells on 7, 9, 11 days post tumor cell seeding. Similarly in 96 well plates after seeding 15,000 cells per well, spheroids were treated with Docetaxel (25–400 µM) on 7, 9, 11 days post cell seeding. The determination of size and number of spheroids was performed on day 9, 11 and 13 using inverted microscope. The alamarBlue® assay was performed to determine number of cells at the end point. The anticancer efficacy was defined in terms of the IC_50_ value based on alamarBlue® assay. Results were compared with 2D culture systems.

### Cytotoxicity of anticancer drugs

Based on the preliminary experiments, it was observed that by 7 d in culture, spheroids attained diameter of >100 µm. Therefore, 7 d was considered as treatment initiation point. Treatment was given at 7 d, 9 d and 11 d. At every treatment time, 24 h drug exposure was followed by a 24 h washout period. Cisplatin, Gemcitabine, 5-Fluorouracil and Camptothecin at concentrations ranges of 25–400 µM were used for the treatment. After the last dose (13 d), the effect of these anticancer drugs on spheroid number and size distribution was studied.

### AlamarBlue® Assays

At 14 d in culture cell viability and metabolic activity was measured using the alamarBlue® assay which is based on the conversion of a non-fluorescent dye to the red fluorescent dye resorufin in response to chemical reduction of growth medium resulting from cell growth. Briefly, 10% alamarBlue® dye with respect to the volume of the medium in each well was added. After one hour of incubation, plates were read for fluorescence intensity at 530 nm & 590 nm wavelength for excitation and emission, respectively. Cells at different densities were incubated with alamarBlue® and after optimum incubation time fluorescence was measured. This standard curve (cell number vs. fluorescence) was utilized to quantify the cell density from the 3D alginate scaffold system.

### Immunohistochemistry

After the drug treatment, spheroids were isolated from the AlgiMatrix™ and spread on the microscopic slide by cytospin. Anticancer drug induced apoptosis was studied by cleaved caspase 3 expression levels by immunohistochemistry (IHC). Cleaved caspase-3 kit was purchased from Cell Signaling Technology and IHC was performed as per the recommended protocol. At the end of the apoptotic stain, counter stain was done by Hematoxylin. Apoptotic positive brown colored cells were visualized with optical microscope and the number of apoptotic cells was counted. Similarly, in 2D cultures, after treatment with drugs, IHC was performed to compare the extent of 2D and 3D cell models to study anticancer drug induced apoptosis.

### RT-PCR

After harvesting the cells or spheroids, RNA was extracted by Trizol reagent (Invitrogen). RNA was quantified by UV method. cDNA was synthesized from the isolated RNA by using suitable mix of nucleotides. After cDNA synthesis, antiapoptotic Bcl-2 mRNA expressions were amplified and quantified by standard RT-PCR protocol.

### Free drug Doxorubicin and nanoparticle uptake studies

Cells were grown in the 6 well 3D alginate scaffold plates at a density of 0.25 million cells/well. Seven days after growing, media was removed and the matrix gel containing the spheroids was dissolved with matrix dissolving buffer. After separating the spheroids from the matrix by centrifugation, spheroids were resuspended in PBS (pH 7.4) and incubated with either free Doxorubicin or DIO oil loaded nanoparticles (NLC). For NLC nanoparticles preparation previously reported method was followed [16]. After 2 hours of incubation, spheroids were washed with PBS twice to remove the unbound free Doxorubicin and NLCs. Finally, spheroids were observed with the fluorescent microscope at excitation and emission wavelength of 470 nm and 585 nm for Doxorubicin and 484 nm and 501 nm for DIO NLCs. Green fluorescence containing spheroid images were captured. In a separate set of experiments after incubating the cells or spheroids with Doxorubicin or DIO NLCs, relative fluorescence was measured by adjusting total cell number. The relative fluorescence units were compared between free Doxorubicin and nanoparticle groups.

### Comparison of 2D and 3D cytotoxicity data to establish 3D-based in vitro tumor model for high throughput screening

Cytotoxicity was determined for Docetaxel, Cisplatin, Gemcitabine, 5-Fluorouracil and Camptothecin in conventional 2D and alginate scaffold-based 3D systems. IC_50_ values obtained from 2D and 3D modes were compared. The comparison between H1650 parental cells and stem cells were done to study the sensitivity of cancer stem cells in 3D culture systems. Similarly, wherever applicable, 2D and 3D data was compared.

### Data analysis and Statistics

Data was represented as mean ± sem. One way ANOVA followed by Tukey's test was used to compare the statistical difference among the groups. P value<0.05 was considered as statistically significant.

## Results

### Development and Optimization of In-Vitro 3D Lung Tumor Model

#### A) 3D alginate scaffold in 6 well plates

**Figure 1:**
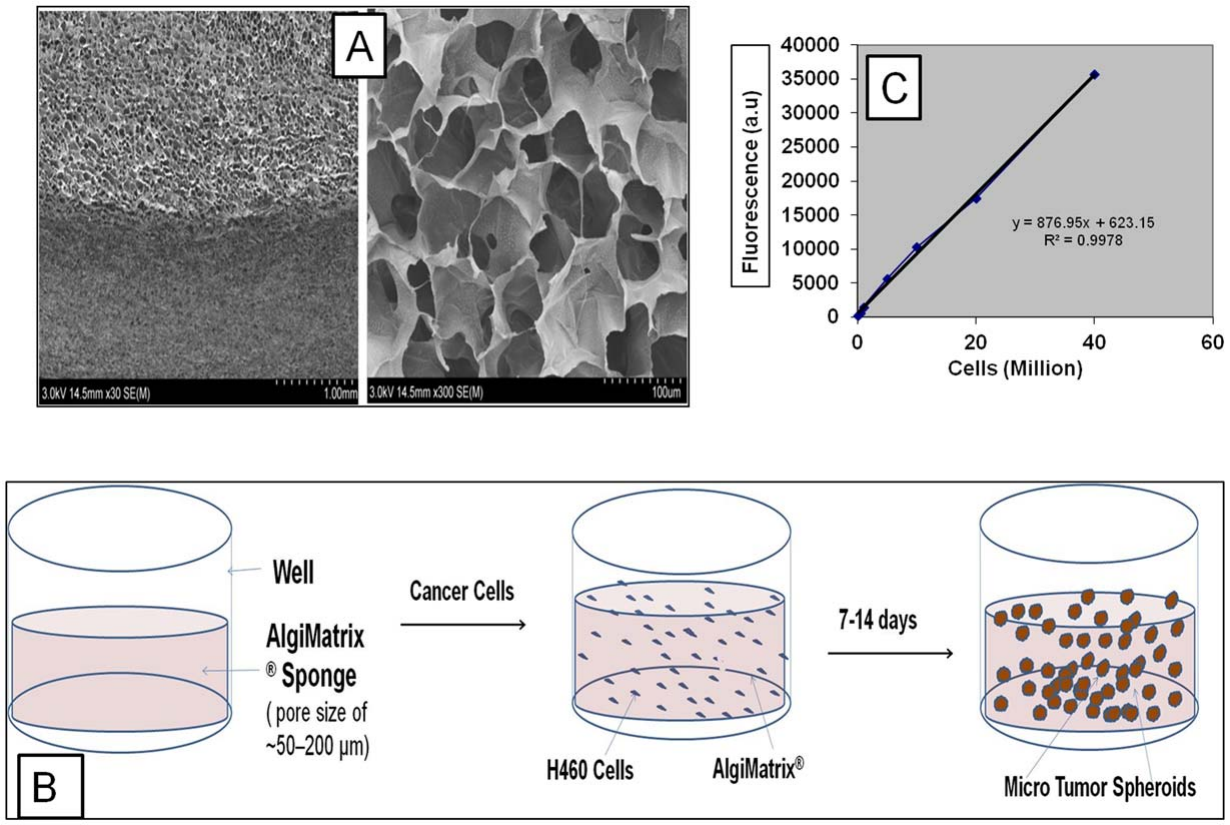
Algimatrix 3D culture system. Scanning Electron Microscopic (SEM) images of 3D alginate scaffolds at lower resolution (left panel, 30 K) and higher resolution (right panel, 300 K), figure shows pore sizes in the matrix to accumulate the cells and grow them as spheroids (A). B) The schematic representation of 3D alginate scaffold wells and how spheroids are formed and grown in the 3D alginate scaffold cell culture system upon seeding the cells into the porous alginate media, C) Standard curve for H460 cells in AlamarBlue assay. Cells at different densities were incubated with AlamarBlue dye, after optimum incubation time point (4 h) fluorescence was measured at 530 and 570 excitation and emission, respectively.

**Figure 2:**
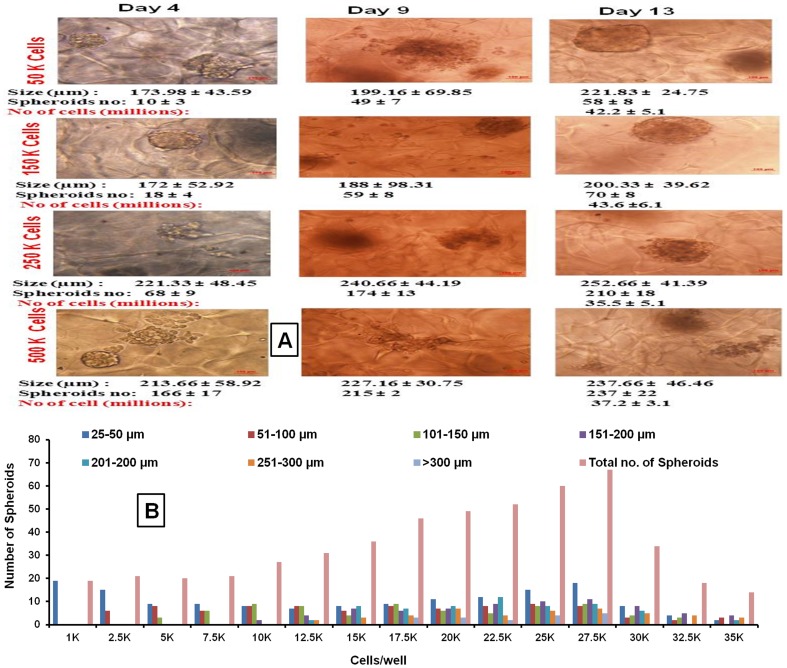
Development and optimization of an in-vitro 3D alginate scaffold system. A) H460 lung tumor model with different cell number over 13 d culture period in a 6 well plate. Figure 2A shows spheroids at 4 d and 13 d days post seeding with different seeding densities (0.05, 0.15, 0.25 and 0.5 million cells/well). Average spheroid size and number were noted at 4 d and 13 d post cell seeding into 3D alginate scaffold. At the end (13 d in culture) total number of cells per well were measured by alamarBlue® assay, B) Graphical and tabular representation of distribution of spheroids by size and seeding density in 96 well plate formats of 3D alginate scaffolds. H460 cells at varying cell density (1–35×10^3^) were introduced into the each 96 plate wells and at the 13^th^ day in vitro tumors in spheroid forms were counted under inverted microscope.

The scanning electron microscopic analysis of 3D alginate scaffold evidenced the distinct pores in matrix which are essential for cells to grow as spheroids (Figure 1A). The porous nature of this system provides the required space for the cells to grow them as in vitro lung tumor models and other organ types based on the types of cells incorporated. Figure 1B shows the schematic representation of spheroid growth in alginate matrix. To calculate the total cell number AlamarBlue assay based standard curve for H460 cells was developed (Figure 1C). This standard curve was utilized to quantify the cell density from the 3D alginate scaffold system. Measurable translucent spheroids were formed as early as 4 days after cell seeding and grew continuously for up to 3 weeks. However, considering the pore size of the matrix and possible development of necrotic regions inside the spheroid due to insufficient oxygen diffusion, these spheroids were allowed to grow for only 2 weeks. Cell titrations for H460, A549, H1650 and H1650 (stem cell) human NSCLC cell lines were done by using different cell concentrations (0.05, 0.10, 0.15, 0.25, 0.50, 0.75, 1.0, 1.25, 1.50, 3 & 6 million cells/well in a 6-well plate) to determine the optimal seeding density and time required for the formation of spheroids. Considering optimal spheroid growth characteristics and size distribution, seeding densities of 0.15×10^6^ & 0.25×10^6^ cells per well were chosen to establish an alginate scaffold 3D tumor model. Average spheroid size at the end of two weeks was found to be 200.33±39.62 µm for 0.15×10^6^ cell density & 252.66±41.39 µm for 0.15×10^6^ cell density, respectively. Figure 2A shows the effect of H460 cell seeding density on spheroid number, average size and total cell number per 6 well plates at 4 d and 13 d post cell seeding. AlamarBlue® assay suggested that total number of cells present after 2 week culture period was 40.68±8.4× and 43.60±6.8×10^6^ for H460 cells seeded with 0.15×10^6^ and 0.25×10^6^ cells (Figure 2A).

In our preliminary studies, morphological and microscopic observations suggested greater proliferation and cell viability of lung cancer cells in 3D systems than the conventional 2D cell culture systems (data not shown). Cells cultured on 3D alginate scaffolds exhibited unique morphology. Based on the size of spheroids (∼250 µm), it was observed that two weeks was the optimum time to culture cells on 3D alginate scaffold. Similar growth behavior was observed for the A549, H1650 lung cancer cells.

#### B) 3D alginate scaffold in 96 well plates

The 3D alginate scaffold H460 lung tumor model using 96 well plates with seeding densities of 1–35×10^3^ cells were studied to optimize the in vitro tumor method for a high throughput screening application. Total number of tumor spheroids increased with cell density up to 27.5×10^3^ cells. Beyond 30×10^3^ cells/well, the spheroid growth characteristics were compromised, which was evidenced by a decrease in the total spheroid number and spheroid size distribution (Figure 2B). Based on these observations, 15×10^3^ was selected as the suitable cell density for further studies in 96 well formats. The development of an alginate scaffold based 3D lung tumor model using 96 well plates will have an advantage over 6 well plates in terms of faster throughput screening of anticancer drugs. Graphical and tabular representation of distribution of spheroids by size and seeding density in 3D alginate scaffold 96 well plate format are shown in Figure 2B. Considering the better spheroid distribution (maximum number of spheroids in the size range of 200–250 µm and fewer number of spheroids >300 µm) the 15×10^3^ cell/well seeding density was selected for the 96 well format anticancer screening.

### Comparison of spheroid growth characteristics with H1650 parental and stem lung cancer cells and effects of anticancer drugs

**Figure 3:**
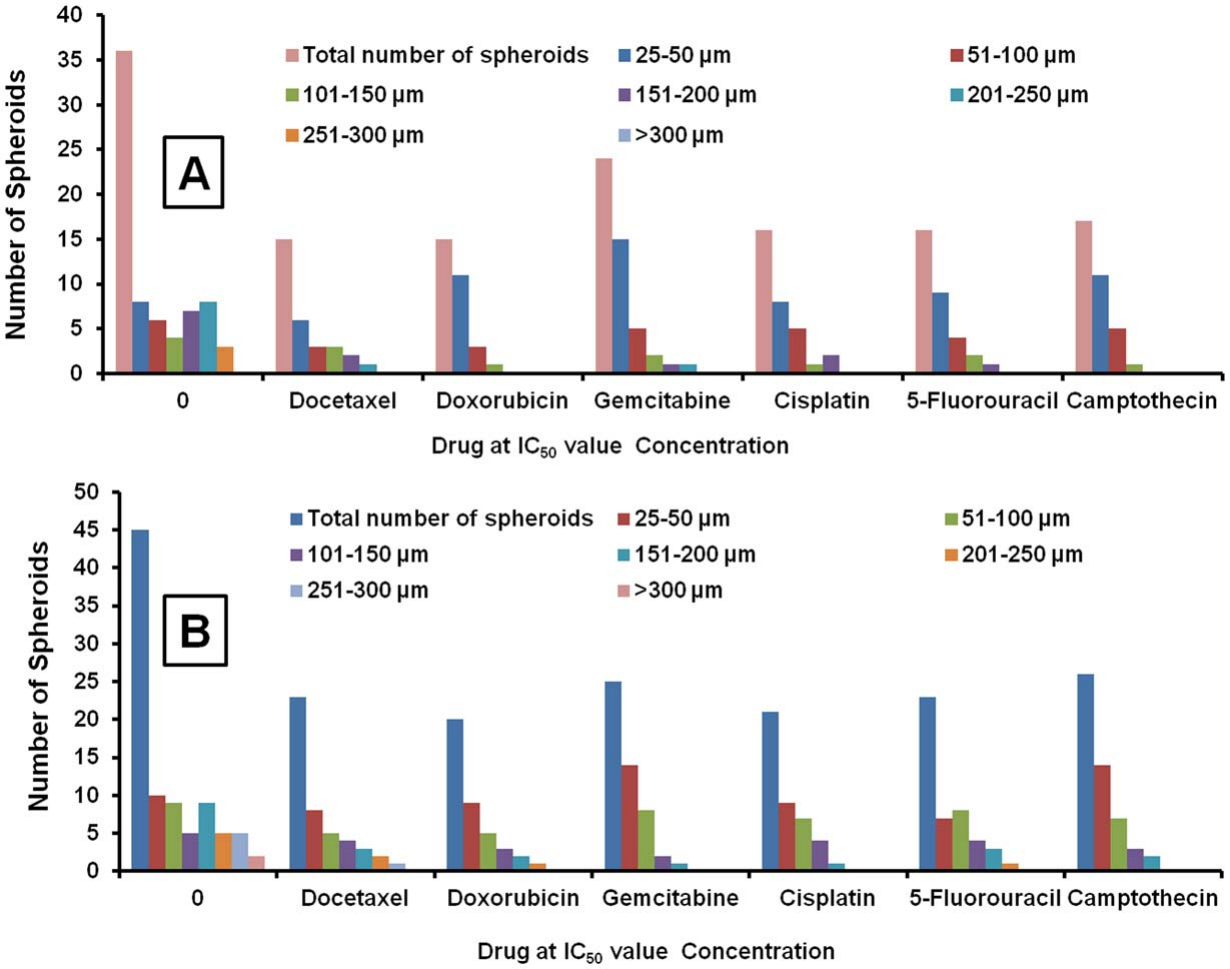
Comparative growth characteristics of spheroids. Total spheroid number and size distribution of H1650 parental and stem cells in 3D algimatrix scaffolds 96 well plate format. The growth characteristics of H1650 parental cells (A) and H1650 stem cells (B) at IC_50_ concentrations of various anticancer drugs (generated on H1650 parental cells).

The growth characteristics of H1650 parental and stem cancer cells with 0.15×10^6^ and 0.25×10^6^ were investigated. After 2 weeks culture period, total number of cells present was 32.6×10^6^ and 39.2×10^6^ for H1650 parental cells seeded with 0.15×10^6^ and 0.25×10^6^ cells. In H1650 stem cells total number of cells were 38.4 ×10^6^ and 42.9×10^6^ in the wells seeded with 0.15×10^6^ and 0.25×10^6^ cells, respectively (Table S1 & S2). In 96 well experiments, the spheroid size distribution pattern and total number of spheroids were characterized. H1650 stem cells exhibited aggressive growth behavior in terms of total number of spheroids and spheroid size distribution. Compared to H1650 parental cells, stem cells generated a greater number of total spheroids (36±4 vs 45±6 µm) and more number of spheroids (3±1 vs 7±2) in the larger size range (>250 µm) (Figure 3A&B). At IC_50_ value concentrations of various anticancer drugs (generated on parental cells), H1650 cancer stem cells exhibited more resistance to reduction in tumor spheroid sizes and total spheroid number compared to H1650 parental cells. The same IC_50_ concentrations in H1650 parental cells resulted in a greater than 50% decrease in the total spheroids, with more spheroids distributed in the lower diameter range (<100 µm) (Figure 3A). Whereas, for H1650 stem cells the total number of spheroids was greater than 50% of the untreated control group (Figure 3B). These observations support the concept of the highly resistant nature of cancer stem cells (CSCs) to chemotherapeutic agents [17].

### Effect of Docetaxel and other anticancer drugs on H460, A549, H1650 parental and H1650 stem cells in 3D alginate scaffold system

**Figure 4:**
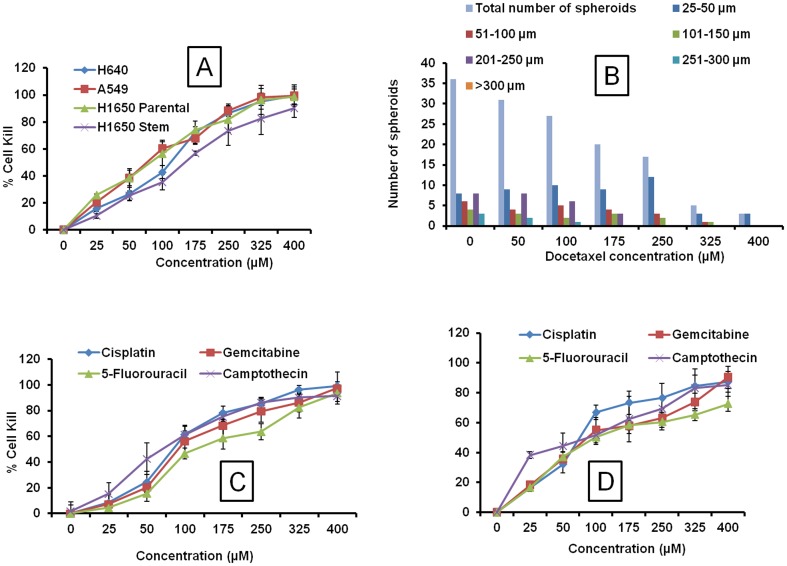
Effect of anticancer agents in 3D system. A) Effect of Docetaxel on H460, A549, H1650 parental and stem lung tumor cells in 3D alginate scaffold system. B) Spheroid size distribution at the various concentrations of Docetaxel on H460 lung cells in 3D alginate scaffold system. Cytotoxicity of anticancer drugs (Cisplatin, Gemcitabine, 5-Fluorouracil and Camptothecin) in 3D alginate scaffold systems on C) H460 and D) A549 lung cancer cells. Each data point is represented as mean ± sem (n = 4–6).

The cytotoxicity data of Docetaxel was compared in 2D and 3D culture plates with H460, A549, H1650 parental and H1650 stem cells. The IC_50_ in 2D experiments was found to be in the range of 1.41, 1.94, 2.70, 14.53 µM for H460, A549 H1650 parental and H1650 stem cells, respectively Table 1. The IC_50_ values for Docetaxel in 3D system were found to be 76.27, 118.11, 81.85 and 151.04 µM for H460, A549, H1650 parental and stem cells, respectively (Figure 4A and Table 1). The higher IC_50_ in stem cells suggested the resistant nature of these cells to chemotherapy. Increased chemoresistance was found in spheroid cultures compared to 2D culture systems. IC_50_ values for the 3D spheroid cultures ranged from 10 to 60-fold higher than their 2D counterparts. The effect of Docetaxel on spheroid size distribution on H460 cells was examined. Graphical and Tabular representation of effect of Docetaxel on spheroid size and total numbers are shown in Figure 3B. Docetaxel produced dose-dependent spheroid size inhibition. At concentrations within the range of 100–175 µM, the total number of spheroids was 50 percent of the total in untreated control wells. With increased drug concentration, the spheroid diameter distributions tended to be lower (25–50 µm) (Figure 4B).

**Table 1:** The comparative IC_50_ values of Docetaxel on H460, A549, H1650 parental and stem lung cells in 2D and 3D alginate scaffold system.

Cell type	IC_50_	
	2D	3D
**H460**	1.41±0.29	76.27±8.52@
**A549**	1.94±0.35	118.11±12.42^@^
**H1650 Parental**	2.70±0.66	81.95±6.34@
**H1650 Stem**	14.53±1.24	151.04±15.73@

Each data point is represented as mean ± sem (n = 3–4).

@ P<0.001 Vs respective 2D groups.

In cytotoxicity assay with H460 lung cancer cells, IC_50_ values in 3D alginate scaffold systems for Cisplatin, Gemcitabine, 5-Fluorouracil and Camptothecin were significantly higher than the 2D culture systems (Figure 4C and Table 2). A549 lung cancer cells produced higher IC_50_ values 75.79, 87.31, 99.17 and 89.74 µM, respectively for Cisplatin, Gemcitabine, 5-Flourouracil and Camptothecin in 3D alginate scaffolds compared to 4.20, 2.56, 3.21 and 1.36 µM, respectively in conventional 2D culture systems (Figure 4D and Table 2). A similar kind of pattern of results were observed for H460, A549 and H1650 cells, with IC_50_ values in 3D systems significantly greater than the 2D culture systems (Table 2). The poor penetration of the chemotherapeutic drugs into the in vitro tumor spheroid might be the reason for the observed resistance, similar kind of environment is seen in vivo tumors [18]. However, in 2D cultures there is possibly lack of penetration barriers.

**Table 2:** Comparative analysis of IC_50_ values of various anticancer drugs in 2D and 3D systems.

Drugs	IC50 value (µM)							
	H460 Cells		A549 Cells		H1650 Parental Cells		H1650 Stem Cells	
	2D	3D	2D	3D	2D	3D	2D	3D
**Cisplatin**	3.47±0.45	84.26±5.63@	4.20±0.2 1	75.79±4.52@	2.09±0.98	66.13±7.36@	4.84±0.62	126.14±12.42@
**Gemcitabine**	2.33±0.16	91.07±7.01@	2.56±0.45	87.31±9.64@	2.68±0.58	103.72±9.68@	6.03±0.84	177.79±14.03@
**5-Fluorouracil**	3.62±0.52	120.94±12.65@	3.21±1.58	99.17±6.24@	2.63±0.37	100.44±8.92@	6.87±0.46	148.31±6.56@
**Camptothecin**	2.59±0.74	69.72±7.82@	1.36±0.17	89.74±7.45@	4.48±0.81	51.84±4.81@	7.49±1.05	95.46±10.68@

Each data point is represented as mean ± sem (n = 4–5).

@ P<0.001 Vs respective 2D groups.

### Comparative cytotoxicity of anticancer drugs on H1650 parental and stem lung cancer cells in 2D and 3D culture systems

**Figure 5:**
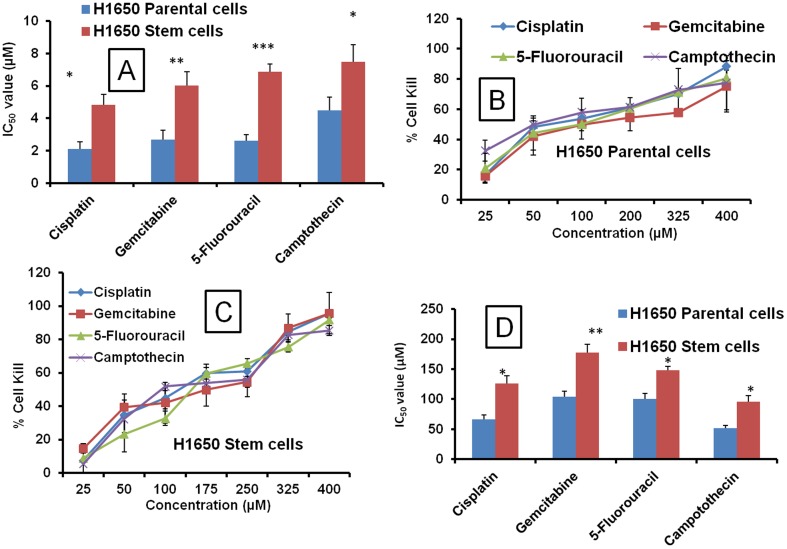
Comparative IC_50_ values of various anticancer drugs in 2D and 3D culture system. A) Cytotoxicity of various anticancer drugs (Cisplatin, Gemcitabine, 5-Fluorouracil and Camptothecin) in 2D H1650 parental and stem cells. B) Cytotoxicity of various anticancer drugs in 3D alginate scaffold systems on H1650 parental, C) H1650 stem cells. D) Comparative cytotoxicity IC_50_ values of various anticancer drugs in 3D alginate scaffold systems on H1650 parental and stem cells. Each data point is represented as mean ± sem (n = 4–6). *<0.05, **P<0.01 and ***P<0.001 Vs respective H1650 parental cell groups.

In 2D culture systems, H1650 cancer stem cells exhibited higher IC_50_ values than the H1650 parental cells, which indicate the resistant nature of cancer stem cells compared to parental cancer cells. The IC_50_ values for H1650 parental cells with Cisplatin, Gemcitabine, 5-Fluorouracil and Camptothecin in 2D systems are 2.09, 2.68, 2.63 and 4.48 µM, respectively. In H1650 stem cells, the IC_50_ values were 4.84, 6.03, 6.87 and 7.49 for Cisplatin, Gemcitabine, 5-Fluorouracil and Camptothecin, respectively (Figure 5A and Table 2). Similarly, 3D models also showed higher IC_50_ values with H1650 cancer stem cells compared to H1650 parental cells (Figure 5B&C). The IC_50_ values for Cisplatin, Gemcitabine, 5-Fluorouracil and Camptothecin were 1.9, 1.71, 1.47 and 1.84, respectively folds higher in H1650 stem cells compared to the H1650 parental cells, indicating the suitability of culturing the cancer stem cells in 3D models to better study their stem cell characteristics (Figure 5D).

### Overall comparison of cytotoxicity between 2D and 3D systems at IC_50_ values

With all cell types tested (H460, A549, H1650 parental and stem cells), a consistent pattern of cytotoxicity profile was observed. As expected there was a significant difference in the cytotoxicity profile of 2D and 3D culture systems. Several fold (5–20) increases in the IC_50_ values were observed in 3D models compared to conventional 2D systems. H1650 lung cancer stem cells demonstrated higher IC_50_ values than the their H1650 parental cell counterparts in both 2D and 3D culture modes, indicating the increased resistance potential of cancer stem cells compared to parental cells (Table 2).

### Doxorubicin uptake by spheroids

**Figure 6:**
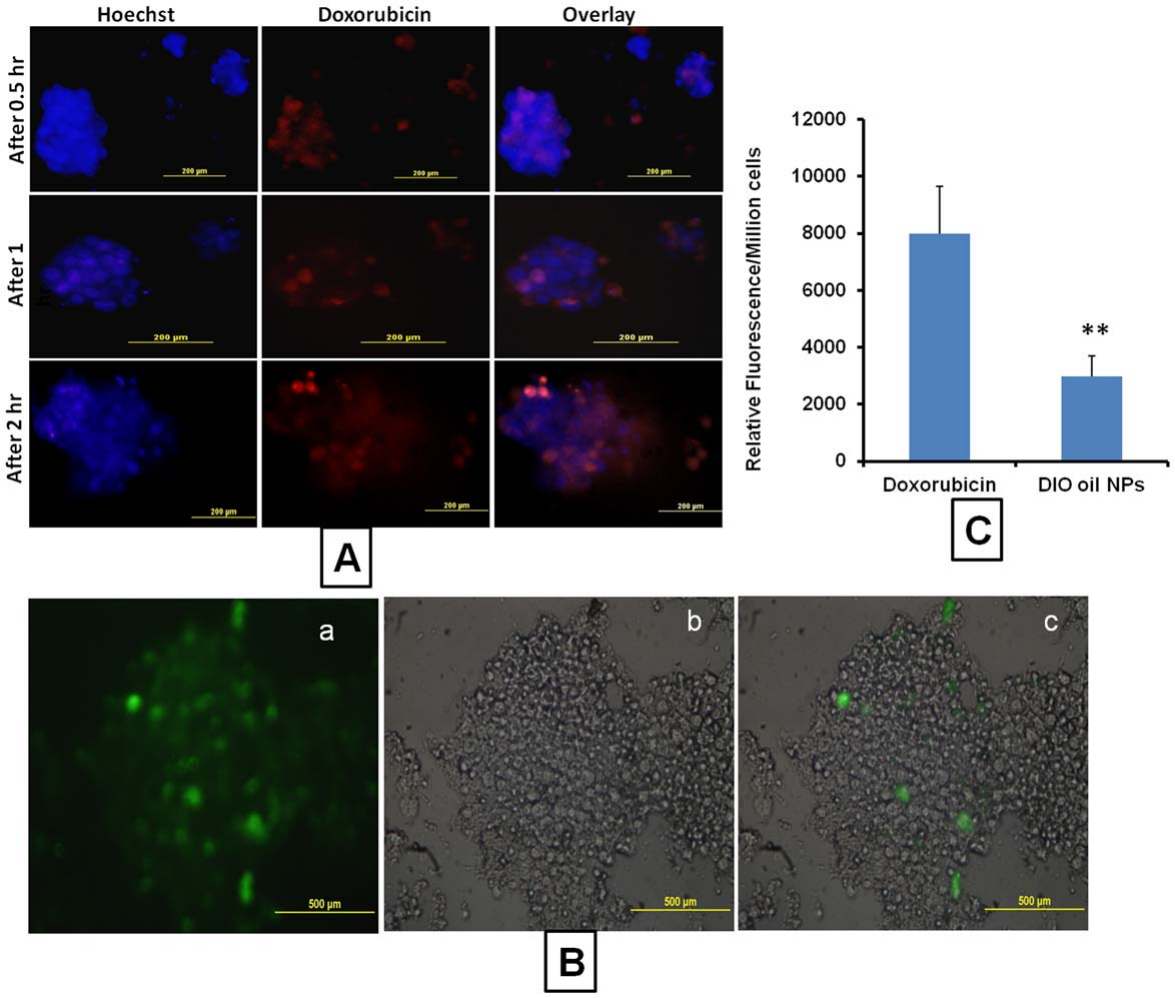
Drug and nanoparticles uptake by spheroids. A) Representative images of Doxorubicin uptake in H1650 parental cell spheroids grown in 3D alginate scaffold system Uptake Images were taken at 0.5, 1 and 2 h time points, B) Representative Images of the nanoparticle uptake by H1650 parental cell spheroids. DIO oil was encapsulated in NLC and incubated with the spheroids for 2 hours, a) Fluorescent image, b) DIC image and c) merged image. The fluorescent images clearly indicates the nanoparticles uptake into spheroids, C) Relative fluorescence intensities of free Doxorubicin uptake and DIO oil loaded NLC nanoparticles into 3D spheroids. Each data point is represented as mean ± sem (n = 3). **P<0.01 Vs Doxorubicin group.

Spheroids were isolated from the matrix and incubated with Doxorubicin. At different time points 0.5, 1, 2 h) the fluorescent intensities were measured. It was found that with time the fluorescent intensities (Doxorubicin uptake) were increased with maximum fluorescence at 2 h incubation period. The mass balance analysis of Doxorubicin inside the spheroid cells and outside the spheroids indicated that only 10.52% of added total Doxorubicin (10 µg) penetrated into the tumor spheroids (Figure 6A).

### Nanoparticle uptake by spheroids

DIO oil loaded nanostructured lipid carrier (NLC) particles were incubated with isolated spheroids. It was found that the penetration of NLCs into the tumor spheroids was very limited, only peripheral cells associated with the spheroids showed NLC uptake (green fluorescence). Whereas cells located inside the spheroid did not show any fluorescence. Further relative fluorescence analysis of fluorescent uptake with free Doxorubicin and DIO oil loaded NLCs indicated that compared to NLCs, free doxorubicin produced 5.56 folds higher fluorescent intensities. That means in 3D tumor spheroids, the uptake of nanoparticles is significantly lesser than the free drug (Figure 6B&C). Similar type of poor intratumoral nanoparticle distribution is expected in vivo tumors due to high fibrous nature [19].

### Immunohistochemistry of 2D and 3D cells for cleaved Caspase 3 expression

**Figure 7:**
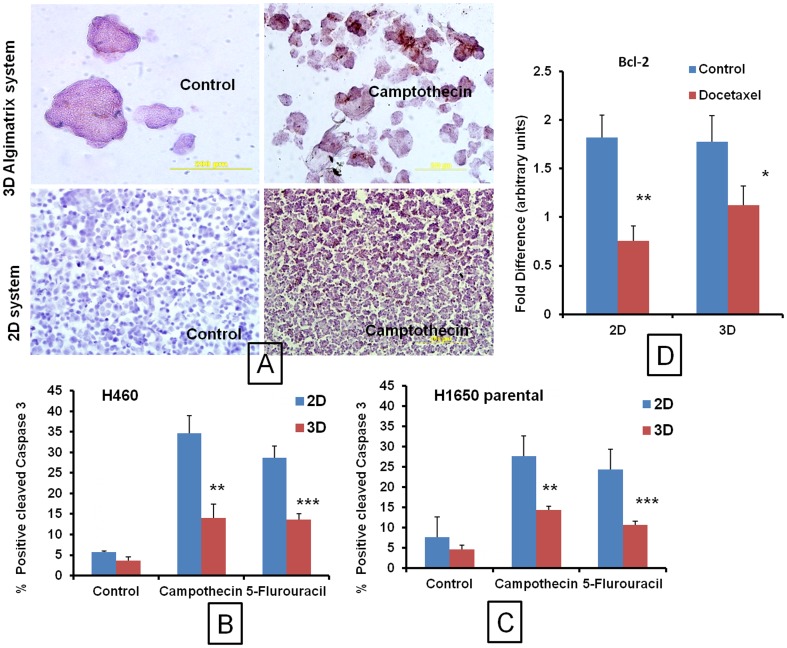
Immunohistochemistry and RT-PCR analysis. A) Representative images of immunohistochemistry of H1650 parental cells or spheroids grown in 2D and 3D culture systems for cleaved caspase positive cells and effect of Camptothecin and 5-Fluorouracil on cleaved caspase expression. Comparative Immunohistochemistry of B) H460 and C) H1650 parental cells or spheroids grown in 2D and 3D culture systems for cleaved caspase 3 positive cells. D) RT-PCR analysis of RNA isolated from 2D and 3D culture systems and effect of Docetaxel on Bcl-2 expression in H460 cells. The IC_50_ concentration of Docetaxel 2D: 1.41 µM and 76.27 µM were used for treatment. Each data point is represented as mean ± sem (n = 3). *<0.05, **P<0.01 and ***P<0.001 Vs respective 2D groups.

Anticancer drug induced apoptosis was studied using cleaved Caspase 3 expression by immunohistochemistry (IHC). In both 2D and 3D cultures, IC_50_ concentrations were used to study apoptosis. Cleaved Caspase 3 positive cells for H460 and H1650 cells were found to be 1.54 and 1.64- folds higher, respectively in 2D models compared to 3D models. After treatment with 5-Fluorouracil and Camptothecin the apoptotic cells were 2.09 and 2.47 folds reduced in 3D cultures compared to monolayer cultures in H460 cells, respectively. A similar pattern of reduced apoptotic cells was observed in H1650 parental cells treated with 5-Fluorouracil and Camptothecin (Figures 7 A–C). The IHC data was in line with the cytotoxicity data. The reduced apoptotic levels in 3D systems even at higher drug concentration further support the hypothesis of poor in vitro tumor penetration and increased drug resistance due to poor spheroid uptake.

### Bcl-2 expression by RT-PCR

The changes in the antiapoptotic mRNA expressions were significantly (2.4 fold) decreased in 2D culture system with Docetaxel treatment at IC_50_ value of 1.41 µM. However, treatment with Docetaxel in 3D culture using the 3D IC_50_ concentration (76.27 µM) resulted in 1.51 fold lesser decrease in the Bcl-2 expression compared to 2D Docetaxel groups (Figure 7D). These results correlate with the cytotoxicity, uptake and IHC studies.

## Discussion

3D cell culture platforms have been a subject of interest since they demonstrate significant differences in cell differentiation and behavior compared to conventional 2D systems [5], [7], [20], [21], [22]. Considering the advantages with 3D cell culture models particularly in cancer research, we have developed an in-vitro lung tumor model by using biologically inert 3D alginate scaffolds (AlgiMatrix™). 3D systems may facilitate the expression of hallmark features of cancer cells, such as unlimited proliferation, self-sufficiency in growth signals, resistance to anti-growth signals, anti-apoptosis mechanisms, invasion, metastasis and angiogenesis [8], [23]. In this study, we report the detailed standardization of in vitro 3D tumor models, comparison of cytotoxicity, drug and nanoparticle uptake and immunohistochemistry and gene expression in traditional monolayer cell culture systems and 3D alginate cell culture systems.

Cells cultured in various 3D systems are well organized with consistent shape and morphology and they maintain cell–cell and cell–matrix interactions [6], [24]. The 3D cell culture models can be successfully employed in performing high throughput screening for cancer drug discovery and development. The co-culture of cancer cells with other cells may provide the paracrine signaling events along with the cell-cell and cell-ECM which are lacking in conventional monolayer cultures. Extracellular matrix mediated signals are lost when cells are cultured ex-vivo on 2D plastic substrates which may be restored using 3D cultures [25]. Recent studies suggest that integrins regulate not only tumor cell proliferation, survival and migration; but they also influence their response to anticancer agents [3]. However, in conventional 2D culture models, all these influences are largely masked [26]. By adapting to in vitro 3D tumor models, these important influences can be readily restored [26]. Therefore, the in vivo tumor environment can be simulated in 3D culture systems.

Functional in vitro tumor models that are representative of in vivo tumor progression have been rapidly evolving and various 3D in vitro tumor models have been already reported [5], [15], [22]. Collagen I hydrogel-based 3D in vitro tumors that are representative of the pre-vascularized stages of in vivo solid tumor progression have demonstrated promising angiogenic potential with a statistically significant upregulation of vascular endothelial growth factor (VEGF)-A gene expression and hypoxia-inducible factor (HIF)-1α factors [6]. In an another 3D model, linker engineered spheroids exhibited characteristics of multi-cellular spheroid morphology, gene expression profile, cell-cell interaction, extracellular matrix secretion, oxygen concentration gradients, and cellular functions [15]. Importantly, these linker-engineered spheroids also displayed a resistance to drug penetration similar to mature MCTS, with dose-dependent extracellular accumulation of the drug [15]. In the current study, we have optimized the cell number and time period required to form in vitro tumors with the sizes ranging from 100–250 µm. We have developed in vitro tumors with 6- and 96-well plate formats. Considering the pore size of the matrix and possible development of necrotic regions inside the spheroid due to insufficient oxygen diffusion, the spheroids were allowed to grow for only 2 weeks. The optimized in vitro tumor spheroid sizes used in the entire study were <300 µm in diameter.

To determine the optimal cell seeding density and time required for spheroid formation, we performed cell titration of human non-small lung cancer cell (NSCLCs) lines by using a range of cell densities (0.05–6×10^6^ cells/well) of H460, A549, H1650 NSCLCs in the 6-well plate format. Considering the better spheroid growth characteristics and spheroid size distribution, the optimized cell concentrations of 0.15 & 0.25×10^6^ cells per well was used to establish the 6-well format 3D tumor model. Further, 0.25×10^6^ cells/well was selected as the final cell seeding density. Two weeks post cell seeding, the spheroid sizes were in the range of 230–260 µm which can be considered as suitable in vitro tumor models without the complications of necrotic lesions. This suggest that there is a greater proliferation and cell viability of lung cancer cells in 3D systems compared to conventional 2D cell culture systems [8]. The initial, spheroid growth patterns suggested that it is feasible to grow cancer cell lines in 3D models, though the pore sizes in AlgiMatrix™ are 100–200 µm, the uncontrolled growth behavior of these cancer cells resulted in the spheroid sizes larger than 200 µm.

To bring the in vitro tumor model system a step closer to high throughput screening, 96-well plate formats were standardized. Different cell seeding densities (1–35×10^3^ cells/well) were studied for the spheroid growth behavior and size distribution. Cell seeding densities greater than 30×10^3^ cells exhibited compromised spheroid growth characteristics. This is expected because the number of pores in the alginate scaffold is fixed so that as more cells were seeded, the number of pores available to accommodate the spheroids were saturated which led to limited space for the spheroids to grow.

Docetaxel cytotoxicity was compared in 2D and 3D culture using H460, A549, H1650 parental and H1650 stem cells. Our initial screening suggested that the concentration ranges used in the 2D culture system were not sufficient to produce significant cytotoxicity in 3D models. Based on this observation, the concentration range of 25–400 µM was employed for the 3D based anticancer screening with Docetaxel. As expected and evidenced in the initial screening, IC_50_ values were significantly higher in the 3D alginate format compared to conventional monolayer cultures. The spheroid distribution analysis suggested that Docetaxel at 100–175 µM concentrations inhibited the total spheroid number by 50% compared to the untreated control. This observation supports the higher resistance of spheroids for standard anticancer drugs.

In line with our assumptions, Cisplatin, Gemcitabine, 5-Flourouracil, Camptothecin, and Docetaxel treatments in 3D culture resulted in higher IC_50_ values than the 2D cell culture models. The phenomenon of increased IC_50_ values and chemoresistance to anticancer drugs has been attributed to several mechanisms, including a decreased penetration of anticancer drugs, increased pro-survival signaling, and/or upregulation of genes conferring drug resistance etc [18], [22]. In a similar fashion, higher survival rates after exposure to the anticancer drug, paclitaxel, were observed in cell spheroids grown in hydrogels compared to cell monolayers in 2D [24]. Thus, 2D evaluation of chemosensitivity may not reflect pathophysiological events seen in patients suggesting that screening in the 3D format may provide information not available in standard 2D experiments.

Side population cancer stem cells (CSCs) originating from tumors are responsible for tumor recurrence and relapse through drug resistance mechanisms [27], [28]. We explored this unique 3D scaffold system to grow the H1650 lung CSCs which are resistant to chemotherapy. In the conventional 2D models, H1650 CSCs require a unique surface coating to grow; however, when grown in 3D culture systems, CSCs cells exhibited very aggressive growth behavior. The numbers of spheroids was higher for stem cells compared to H1650 parental cells. CSCs also demonstrated larger spheroids (>250 µm) on average in the 3D culture system. We have studied the H1650 cancer stem cells cytotoxicity in 3D culture and found that the IC_50_ values are higher in 3D culture systems compared to 2D culture systems. Cisplatin, Gemcitabine, 5-Flourouracil and Camptothecin treatment in 3D alginate culture resulted in higher IC_50_ values than the 2D IC_50_ values. This observation is in line with our results with other cell lines. The higher IC_50_ values for 3D cell cultures indicate the possible resistance acquired by the lung cancer cells while growing in the 3D scaffold which closely mimics the in vivo tumor environment. Our results are in agreement with the various spheroid based anticancer evaluations with decreased sensitivity to the chemotherapeutic agents [29], [30]. In 3D cultures, H1650 CSCs exhibited higher IC_50_ value than the H1650 parental cells, which indicates the resistant nature of cancer stem cells compared to normal cancer cells. At IC_50_ concentrations (generated on parental cells) of various anticancer drugs, H1650 cancer stem cells exhibited more resistance to decreases in tumor spheroids size and total number compared to H1650 parental cells. These observations support the theory that CSCs possess high resistance to chemotherapeutic agents [27], [28].

CSCs grown in 2D culture may lose their stem cell properties, whereas CSCs grown in the 3D system may further increase the drug resistance. This was explained by higher concentrations of anticancer drugs being needed in 3D alginate cultures compared to 2D models. The increased IC_50_ values support the recently reported 3D matrix driven CSCs hypothesis in which 3D scaffold culturing of MCF-7 breast cancer cells resulted in high tumorigenesis and increased chemoresistance to anticancer drugs [31]. Further, 3D scaffold cultures resulted in the diversification of cell morphologies and increased cell proliferation. Pro-angiogenic growth factors and the transcriptions of matrix metalloproteinases (MMPs) were significantly increased in reported 3D systems [31]. In addition, cells cultured in this 3D system resulted in the increased CSCs population. Increased expression of stem cells characteristic markers EMT, CD44, SOX-2 OCT4A suggested the existence of CSCs. In the same study the CSCs properties were further confirmed by high tumorigenicity in vivo with xenografts from 3D cells formed larger tumors [31].

To understand this disparity in cytotoxic responses of standard chemotherapeutic drugs in 2D and 3D based culture systems, we studied the penetration efficiency of free drug Doxorubicin and nanoparticle penetration into the spheroids. Doxorubicin uptake studies suggested that spheroids uptake only a fraction of the available drug. It was shown that only 10.52% of Doxorubicin was present inside the spheroid. This observation supports the poor penetrating nature of tumor spheroids [8], [10], [32]. Due to insufficient penetration, a high concentration of anticancer drug is required to show cytotoxicity [14], [18]. A similar pattern of poor penetration was reported in another linker-engineered spheroids 3D model system [15]. Doxorubicin uptake studies support the cytotoxicity data observed in this study. The poor penetration could account for the decreased cytotoxicity that was observed in the 3D alginate cultures in this study [8]. The nanoparticle-based nanostructured lipid carrier (NLC) penetration into tumor spheroid**s** demonstrated that a very limited amount (3.41%) of added DIO oil-loaded NLCs penetrated into the spheroid. Microscopic analysis of the NLCs suggested that NLCs were able to penetrate only peripheral regions of the spheroid. The poor penetration of free drug and nanoparticles might be due to increase in the number of tight junctions in the core area of spheroids and increased expression of E-Cadherin a protein involved in the assembly and sealing of tight junctions [33]. Hence, the poor penetrating nature exhibited by spheroids demonstrate very limited nanoparticle uptake. Similarly, in vivo tumors have also been shown to exhibit poor nanoparticle penetration due to the presence of their highly fibrous nature [19], [34]. Hence our results suggest that the 3D alginate culture system may be suitable to mimic the poorly penetrating tumors such that the effect of fibrolytic agents on nanoparticle intratumoral distribution can be studied based using this model. Tumor invasion and metastasis is greatly dependent on the balance between proteolytic and anti-proteolytic activities inside the tumor environment. The metastasis and invasion of cancer cells involve a coordinated degradation and reconstitution of the surrounding extracellular matrices [35]. By virtue of the algimatrix based large spheroid formation, it is expected that suitable environment is created in 3D spheroids to study the role of extracellular matrix and matrix metalloproteinases (MMPs) in in-vitro tumor invasion and metastasis.

The immunohistochemical analysis of spheroids for cleaved caspse-3 (an apoptotic marker) levels suggested significant reduction in the cleaved caspase- 3 levels compared to 2D culture systems. The apoptosis mediated cell death induced by anticancer drugs is significantly decreased in 3D culture systems. This observed phenomenon is possibly due to poor penetration of anticancer drugs into the spheroid [8], [18], [32]. Other proposed mechanism is altered gene expression profiles of cancer cells in 3D tumor models to attain drug resistance [30], [36], [37]. RT-PCR analysis of antiapoptotic marker Bcl-2 further supported that the decreased apoptosis in 3D cells is because of the increased expression of antiapoptotic markers and decreased levels of cleaved Caspase-3. Our observations are in line with the other reported 3D based systems with decreased apoptosis due to altered molecular expressions [10], [36], [38], [39]. Therefore, our cytotoxicity, drug and nanoparticle uptake and molecular studies consistently demonstrate the increased resistance of 3D culture systems compared to conventional cell culture systems. Our three-dimensional model may reflect a more clinically relevant in vitro setting for cancer research. A well characterized 3D model may be useful for rapid screening of a large number of anticancer agents during the drug discovery phase [8]. We speculate that the in vitro tumors developed here may be useful to study various tumor targeting antibodies and formulations.

## Conclusion

The in vitro AlgiMatrix™ 3D lung tumor model was successfully developed using optimized seeding density of NSCLC cells for 6- and 96-well plates. These results strongly support that the 3D alginate scaffold may be used as an in vitro tumor model. Hence, it is possible to predict the anticancer effect of various drugs and formulations in a better fashion than conventional monolayer cultures. As a result, the ability to predict outcomes in preclinical animal models and clinical trials can be better understood because the positive anticancer effects observed in 2D cultures often fail during in vivo testing. The results from our studies are useful to develop a high throughput in vitro tumor model to study the effect of various anticancer agents and various molecular pathways affected by the anticancer drugs and formulations. Collectively, the results from the in vitro tumor models suggest that AlgiMatrix™ 3D platform is a potentially useful predictive tool in cancer biology and anticancer screening. We conclude that 3D cell culture scaffolds such as AlgiMatrix™ serve as a foundation for the creation of more physiologically relevant culture systems for various biomedical applications, particularly in cancer biology. In the future, these 3D spheroid cultures can be expanded to various fields of drug discovery, development and novel drug delivery approaches. In future perspectives, in vitro-in vivo tumor model correlation studies and fabrication of co-culture models are underway to further explore this promising 3D system for cancer research.

“Regulatory Statement: For Research Use Only. Not for use in diagnostic procedures”

## Supporting Information

Table S1 **Growth characteristics of H1650 cancer parental cells in 6 well plate format at 4 d, 9 d and 13 d post cell seeding with 015 and 0.25 million cells/well.** The average spheroid size, total spheroid number/well and total number of spheroids/plate. Each data point is represented as mean ± sem (n = 3).
(DOCX)

Table S2 **Growth characteristics of H1650 cancer stem cells in 6 well plate format at 4 d, 9 d and 13 d post cell seeding with 015 and 0.25 million cells/well.** The average spheroid size, total spheroid number/well and total number of spheroids/plate. Each data point is represented as mean ± sem (n = 3).
(DOCX)
