# Supplementary material for: AlgiMatrix™ Based 3D Cell Culture System as an In-Vitro Tumor Model for Anticancer Studies
Source: PLoS One. 2013 Jan 18;8(1):e53708. doi: 10.1371/journal.pone.0053708 (PMC3548811; doi:10.1371/journal.pone.0053708)
Supplement: Table S1 — Growth characteristics of H1650 cancer parental cells in 6 well plate format at 4 d, 9 d and 13 d post cell seeding with 015 and 0.25 million cells/well. The average spheroid size, total spheroid number/well and total number of spheroids/plate. Each data point is represented as mean ± sem (n = 3). (DOCX) [file pone.0053708.s001.docx]

| **Time Points**  **(Days)** | **Average size of spheroids**  **(µm)** | | **Total Number of spheroids/well** | | **Total Number of Cells (10^6^)/6 well plate** | |
| --- | --- | --- | --- | --- | --- | --- |
|  | 0.15x10^6^ | 0.25x10^6^ | 0.15x10^6^ | 0.25x10^6^ | 0.15x10^6^ | 0.25x10^6^ |
| **4** | 107.7 ± 21.4 | 129.9 ± 25.3 | 47 ± 4 | 64 ± 9 | --- | --- |
| **9** | 185.3 ± 48.2 | 209.9 ± 42.9 | 82 ± 7 | 162 ± 12 | --- | --- |
| **13** | 224.7 ± 59.7 | 251.4 ± 61.5 | 142 ± 8 | 184 ± 12 | 32.6 ± 5.4 | 39.2 ± 4.7 |

**Supplementary Table I.** Growth characteristics of H1650 cancer parental cells in 6 well plate format at 4d, 9d and 13d post cell seeding with 015 and 0.25 million cells/well. The average spheroid size, total spheroid number/well and total number of spheroids/plate. Each data point is represented as mean ± sem (n=3).
