# Supplementary material for: AlgiMatrix™ Based 3D Cell Culture System as an In-Vitro Tumor Model for Anticancer Studies
Source: PLoS One. 2013 Jan 18;8(1):e53708. doi: 10.1371/journal.pone.0053708 (PMC3548811; doi:10.1371/journal.pone.0053708)
Supplement: Table S2 — Growth characteristics of H1650 cancer stem cells in 6 well plate format at 4 d, 9 d and 13 d post cell seeding with 015 and 0.25 million cells/well. The average spheroid size, total spheroid number/well and total number of spheroids/plate. Each data point is represented as mean ± sem (n = 3). (DOCX) [file pone.0053708.s002.docx]

| **Time Points**  **(days)** | **Average size of spheroids**  **(µm)** | | **Total Number of spheroids/** | | **Total Number of Cells (10^6^)**/**6 well plate** | |
| --- | --- | --- | --- | --- | --- | --- |
|  | **0.15x10^6^** | **0.25x10^6^** | **0.15x10^6^** | **0.25x10^6^** | **0.15x10^6^** | **0.25x10^6^** |
| **4** | 177.8 ± 61.7 | 220.7± 61.2 | 48 ± 4 | 77 ± 8 | --- | --- |
| **9** | 205.3 ± 78.1 | 232.9 ± 82.8 | 90 ± 8 | 189 ± 15 | --- | --- |
| **13** | 232.2 ± 89.1 | 263.9 ± 86.4 | 153 ± 18 | 228 ± 17 | 38.4 ± 4.9 | 42.9 ± 3.3 |

**Supplementary Table II.** Growth characteristics of H1650 cancer stem cells in 6 well plate format at 4d, 9d and 13d post cell seeding with 015 and 0.25 million cells/well. The average spheroid size, total spheroid number/well and total number of spheroids/plate. Each data point is represented as mean ± sem (n=3).
